# Green tea extract suppresses airway inflammation via oxidative stress-driven MAPKs/MMP-9 signaling in asthmatic mice and human airway epithelial cells

**DOI:** 10.3389/fimmu.2024.1362404

**Published:** 2024-04-30

**Authors:** Jeong-Won Kim, Jin-Hwa Kim, Ji-Soo Jeong, Chang-Yeop Kim, Eun-Hye Chung, Sung-Hwan Kim, Eui-Ju Hong, Hyo-Jung Kwon, Je-Won Ko, Tae-Won Kim

**Affiliations:** ^1^ College of Veterinary Medicine (BK21 FOUR Program), Chungnam National University, Daejeon, Republic of Korea; ^2^ Jeonbuk Department of Inhalation Research, Korea Institute of Toxicology, Jeongeup, Republic of Korea

**Keywords:** asthma, green tea extract, inflammation, matrix metalloproteinase-9, mitogen-activated protein kinase signaling, oxidative stress

## Abstract

**Introduction:**

The anti-inflammatory effect of green tea extract (GTE) has been confirmed in asthmatic mice, however, the pharmacological mechanism is not fully elucidated.

**Methods:**

To investigate the therapeutic efficacy of GTE in asthma and identify specific pathways, murine model of allergic asthma was established by ovalbumin (OVA) sensitization and the challenge for 4 weeks, with oral treatment using GTE and dexamethasone (DEX). Inflammatory cell counts, cytokines, OVA-specific IgE, airway hyperreactivity, and antioxidant markers in the lung were evaluated. Also, pulmonary histopathological analysis and western blotting were performed. *In vitro*, we established the model by stimulating the human airway epithelial cell line NCI-H292 using lipopolysaccharide, and treating with GTE and mitogen-activated protein kinases (MAPKs) inhibitors.

**Results:**

The GTE100 and GTE400 groups showed a decrease in airway hyperresponsiveness and the number of inflammatory cells in the bronchoalveolar lavage fluid (BALF) compared to the OVA group. GTE treatment also reduced interleukin (IL)‐13, IL-5, and IL‐4 levels in the BALF, and OVA-specific immunoglobulin E levels in the serum compared to those in the OVA group. GTE treatment decreased OVA-induced mucus secretion and airway inflammation. In addition, GTE suppressed the oxidative stress, and phosphorylation of MAPKs, which generally occurs after exposure to OVA. GTE administration also reduced matrix metalloproteinase‐9 activity and protein levels.

**Conclusion:**

GTE effectively inhibited asthmatic respiratory inflammation and mucus hyperproduction induced by OVA inhalation. These results suggest that GTE has the potential to be used for the treatment of asthma.

## Introduction

Allergic asthma is a chronic pulmonary disease characterized by mucus hyperproduction and inflammation, and its prevalence has been gradually increasing (1). T helper 2 (Th2) cells exert key roles in pathogenesis of asthma by secreting Th2 cytokines including interleukin (IL)-4, IL-13, and IL-5 (2). Th2 cytokines not only assemble eosinophils and induce their activation and infiltration but also activate B cells to produce allergen-specific immunoglobulin (Ig) E (3). Considering transduction of signaling molecules, oxidative stress plays crucial roles in the development of asthma. An imbalance between extracellular oxidative inducers and intracellular antioxidant responses produces reactive oxygen species (ROS), leading to the activation of inflammatory signaling. Of these, matrix metalloproteinase (MMP)‐9 and mitogen-activated protein kinases (MAPKs) play a pivotal role in allergic asthma development (4, 5). Previous studies have demonstrated that oxidative stress-driven MAPKs phosphorylation leads to the upregulation of MMP-9 expression, resulting in inflammation and airway remodeling both *in vivo* and in clinical trials (6–8). Therefore, inhibition of these signals may be an important therapeutic target for asthma.

*Camellia sinensis* (L.) Kuntze (green tea) is one of the most popular natural products with numerous health benefits (9). Green tea has antibacterial, antioxidant, anticancer, and anti-inflammatory properties (9, 10). According to previous studies, green tea reduced oxidative and inflammatory damage to the airways of guinea pigs and mediated the inhibition of IgE levels in patients with allergic asthma (11, 12). It contains catechins such as epicatechin, epigallocatechin (EGC), EGC-3-gallate (EGCG), and epicatechin-3-gallate (13). These catechins are the main antioxidants in green tea and exhibit a strong ability to reduce ROS and nitrogen species (13). EGCG, a major component of green tea extract (GTE), reduced mucus production and p38/MMP-9 protein levels in a mouse model of asthma (14). In addition, the protective effects of epicatechin gallate against ovalbumin (OVA)-induced asthma have been confirmed in several *in vivo* studies through the modulation of cytokine expression, T cell populations, and various molecular signaling pathways (15, 16). Based on these results, it is possible that green tea exerted a protective effect in an OVA-induced mouse model of asthma.

To investigate the therapeutic mechanism of GTE in allergic asthma, we examined the levels of mucus secretion, inflammatory cells, and cytokines. Additionally, we performed molecular biology analyses, focusing on the role of GTE in regulating the oxidative stress-mediated MAPKs/MMP-9 signaling pathway.

## Materials and methods

### GTE preparation

Dried green tea powder was purchased from DAJAYEON Co. Ltd (Sacheon, Gyeongnam, Republic of Korea). The source is leaves of *C. sinensis* (L.) Kuntze and the place of production is Sacheon. The plant name was confirmed using “The Plant List” (http://www.theplantlist.org) (Aug 25, 2023). GTE was prepared as described previously (10). The extraction yield was approximately 32%, and GTE contained 36%, 22%, and 7% of catechin, EGCG, and EGC, respectively, as confirmed by HPLC with a UV detector (10).

### *In vivo* experiments

#### Animals and environmental conditions

All experimental procedures were approved by the Institutional Animal Care and Use Committee of Chungnam National University (Approval no. 202206A-CNU-121; June 29, 2022) and were conducted in accordance with the Guidelines for Animal Experiments of Chungnam National University. The research was conducted in accordance with internationally accepted principles for laboratory animal use and care. Male BALB/c mice (7 weeks old, body weight 20–22 g) were supplied by Orient Bio (Gyeonggi-do, Republic of Korea) and allowed to adapt for 8 days. Mice were housed in standard conditions (humidity 50 ± 10%, temperature 23 ± 3°C, 13–18 air changes/h, and 12 h light/dark cycles). Commercial rodent chow and tap water were provided *ad libitum*.

#### Experimental groups

The mice were divided into five groups (*n* = 8/group) as follows (1): normal control (NC), orally treated with phosphate-buffered saline (PBS) from day 16 to day 24 and PBS sensitization/challenge; (2) OVA: orally treated with PBS from day 16 to day 24 and OVA sensitization/challenge; (3) dexamethasone (DEX): orally treated with DEX (3 mg/kg/day) from day 16 to day 24 and OVA sensitization/challenge; and (4) GTE100 and GTE400: orally treated with GTE (100 or 400 mg/kg/day, respectively) from day 16 to day 24 and OVA sensitization/challenge. OVA challenge was conducted as described by earlier (17). All mice were sacrificed under anesthesia two days after the last treatment, and lung tissue and blood were harvested.

#### Airway hyperresponsiveness and evaluation of bronchoalveolar fluid

Airway hyperresponsiveness (AHR) of mice was evaluated by whole-body plethysmography (Allmedicus, Gyeonggi-do, Republic of Korea) at day 1 after the third OVA inhalation as described previously (17). The enhanced pause (Penh) unit was used to represent the data.

To obtain bronchoalveolar lavage fluid (BALF), the mice were euthanized by carbon dioxide inhalation two days after the last OVA inhalation. BALF sampling was performed as described previously (17). The Diff-Quik^®^ staining reagent (Sysmex Corporation, Kobe, Japan) was used for inflammatory cell staining in BALF.

ROS levels in BALF cells were examined using a cellular ROS assay kit (Abcam, Cambridge, UK) in compliance with the manufacturer’s instructions. BALF cells were observed under a confocal microscope (Leica Microsystems, Wetzlar, Germany).

#### Enzyme-linked immunosorbent assay

The levels of IL-13, IL-5, and IL-4 in BALF were assessed using enzyme-linked immunosorbent assay (ELISA) kits (R&D Systems, Oxford, United Kingdom) in accordance with the manufacturer’s instructions. The absorbance was measured at 450 nm using a microplate reader (iMark™; Bio-Rad Laboratories, Hercules, CA).

ELISA kits (BioLegend, San Diego, CA) were used to measure the total IgE and OVA-specific IgE levels in the serum. After the assay procedures, the plates were washed with PBS, and 200 μL of o-phenylenediamine dihydrochloride (Sigma-Aldrich, St. Louis, MO) was added to each well. The absorbance was measured at 450 nm using a microplate reader (Bio-Rad Laboratories).

#### Histopathological/immunohistochemical examination

Lung tissues were processed as described previously (17). To evaluate mucus production and airway inflammation, lung tissue was stained with periodic acid-Schiff (PAS; IMEB Inc. San Marcos, CA) or hematoxylin and eosin (H&E; BBC Biochemical, Mount Vemon, WA). The score of mucus production was calculated as the percentage of purple (positive) stained cells in the total cell count. MMP-9 protein was stained using an immunohistochemistry (IHC) kit (Abcam) following the manufacturer’s instructions. A primary antibody against MMP-9 (1:300; Cell Signaling Technology, Danvers, MA) was used for IHC staining. Brown stained cells are positive for MMP-9. Quantitative analyses were conducted with a light microscope in a blinded manner (Leica Microsystems) at 10× and 20× objective lenses using the IMT i-Solution software (Vancouver, North Road Burnaby, Canada).

#### Western blot

The frozen liver tissues were homogenized with tissue lysis/extraction (Sigma-Aldrich) containing a protease/phosphatase inhibitor cocktail (Sigma-Aldrich) and centrifuged at 10,000 *× g* for 10 min at 4°C to isolate the proteins in the lysate. To examine the levels of proteins related to the MAPKs pathway, we conducted immunoblotting according as described previously (17). Antibodies and dilutions were as follows: total (t)-jun N-terminal kinase (JNK) (1:1,000 dilution), phospho (p)-JNK (1:1,000 dilution), t-p38 (1:1,000 dilution), p-p38 (1:1,000 dilution), t-extracellular signal-regulated kinase (ERK) (1:1,000 dilution), p-ERK (1:1,000 dilution), MMP-9 (1:1,000 dilution), and β-actin (1:2,000 dilution), all obtained from Abcam. Each protein band was photographed using ChemiDoc (Bio-Rad Laboratories).

#### Evaluation of MMP-9 activity in lung tissue

Lung samples were used to evaluate MMP-9 activity. Gelatin zymography was performed as described by (18). For electrophoresis, gels containing 1% gelatin and 10% sodium dodecyl sulfate–polyacrylamide gel electrophoresis were used as MMP substrates. The gels were then washed and incubated for 24 h at 37°C in a developing buffer. The gels were stained with coomassie brilliant blue and destained using 25% methanol and 8% acetic acid in distilled water. The white band on a blue background indicates the activity of MMP-9.

#### Determination of antioxidant markers in lung tissue

Malondialdehyde (MDA), glutathione reductase (GR), glutathione (GSH), superoxide dismutase (SOD), and catalase (CAT) were detected in the lung tissues of mice from all five groups. MDA, GSH, CAT, GR, and SOD assay kits were purchased from Cayman Chemicals (Ann Arbor, MI) and performed in strict accordance with the manufacturer’s protocols. Absorbance (450 nm) was measured using a microplate reader (Bio-Rad Laboratories).

### *In vitro* experiments

#### Cell viability and assessment of cytotoxicity

The human airway epithelial cell line NCI-H292 was cultured in RPMI 1640 medium containing fetal bovine serum and antibiotics (5% CO_2_, 95% air, 37°C). An EZ-Cytox Cytotoxicity Assay Kit (DoGenBio Co., Ltd., Seoul, Republic of Korea) was used to measure cell viability in response to GTE. The NCI-H292 cells were cultured in 96-well plates (2.0 × 10^4^ cells/well, 100 µL), and incubated overnight. The cells were incubated with GTE (between 5 μg/mL to 160 μg/mL, 2-fold increases) and EGCG (Sigma-Aldrich, between 5 μg/mL to 160 μg/mL, 2-fold increases) for 24 h. After GTE or EGCG treatment, the kit solution was inoculated into each well and the plate was incubated for 1 h. Cytotoxicity was evaluated by measuring absorbance (450 nm) using a microplate reader (Bio-Rad Laboratories). Cytotoxicity was normalized to that of control cells.

#### Evaluation of mRNA expression levels

The cells were incubated with 10, 20, and 40 μg/mL of GTE or 20 μg/mL of EGCG for 15 h, and then, inoculated with 0.5 μg/mL lipopolysaccharide (LPS) for 4 h. Total RNA was isolated utilizing the SmartGene Total RNA Extraction Kit (SJ Bioscience, Daejeon, Republic of Korea) according to manufacturer’s instruction. First-strand cDNA was synthesized from 1000 ng of total RNA using a Compact cDNA Synthesis Kit (SJ Bioscience). Quantitative PCR was performed using the CFXTM Connect Real-Time System (Bio-Rad Laboratories) and SYBR Green Q-PCR Master Mix with Low Rox (SJ Bioscience). The relative quantification values of the targets were normalized to those of the housekeeping and untreated controls.

#### Western blot

NCI-H292 cells were incubated with GTE (10, 20, and 40 μg/mL), EGCG (20 μg/mL), and MAPKs inhibitors, including SB2035880 (p38 inhibitor, 10 uM; Sigma-Aldrich), SP600125 (JNK inhibitor, 10 uM; Sigma-Aldrich), and PD098059 (ERK inhibitor, 10 uM; Sigma-Aldrich) for 13 h, followed by treatment with 0.5 μg/mL LPS for 1 day. Cellular proteins were extracted using a RIPA buffer (Sigma–Aldrich) containing a protease/phosphatase inhibitor (Sigma–Aldrich). The experimental methods and antibodies used were identical to *in vivo* experiments.

#### Statistical analysis

The results were expressed as the mean ± standard deviation. To calculate statistical significance, a one-way analysis of variance followed by post-hoc Tukey’s honest significant difference test was used. All calculations were performed using GraphPad InStat (version 3.0; GraphPad Software, Inc., CA). Significance was indicated by a *p* value less than 0.05 or 0.01.

## Results

### *In vivo* experiments

#### Effects of GTE on AHR in asthmatic mice

**Figure 1:**
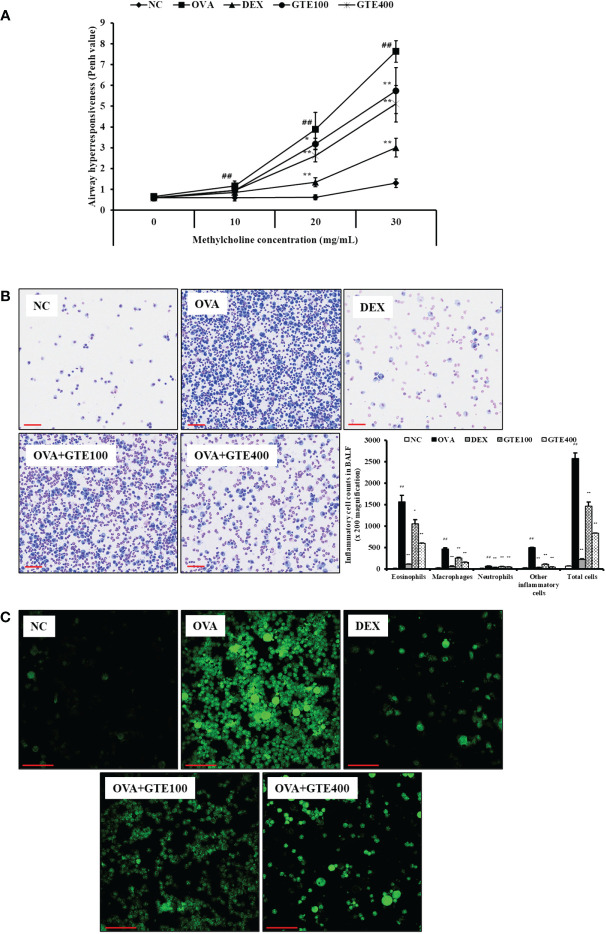
Green tea extract (GTE) decreases airway hyperresponsiveness **(A)**, inflammatory cells in bronchoalveolar lavage fluid **(B)**, and ROS production **(C)**. Values are shown as means ± SD (*n* = 8). Significant differences: ^##^ *p* < 0.01 vs. NC; ^*, **^ *p* < 0.05 and *p* < 0.01 vs. OVA, respectively. Scale bar = 100 µm.

The Penh value of the OVA group was significantly higher than that of the NC group at all methylcholine concentrations (**Figure 1A**). The GTE100 and GTE400 groups treated with 30 mg/mL methylcholine, and the GTE400 group treated with 20 mg/mL methylcholine showed decreased Penh values compared with the OVA group (*p* < 0.01). The Penh values of the DEX group were significantly lower than those of the OVA group treated with 20 and 30 mg/mL methylcholine (*p* < 0.01).

#### Preventive effects of GTE on inflammatory cell counts and ROS production in BALF

To assess the effects of GTE on inflammatory cells in the BALF, neutrophils, lymphocytes, macrophages, and total cell counts were observed. As shown in **Figure 1B**, the inflammatory cell count of the OVA group was significantly higher than that of the NC group (*p* < 0.01). In contrast, the DEX group exhibited a marked decrease in the number of inflammatory cells compared to the OVA group (*p* < 0.01). The GTE-treated mice (100 and 400 mg/kg/day) had a reduced number of inflammatory cells compared to the OVA group (*p* < 0.01).

To evaluate the effect of GTE on ROS production, inflammatory cells were treated with DCFDA. As shown in **Figure 1C**, ROS level was markedly elevated in the OVA group compared with those in the NC group. In contrast, the GTE100 and GTE400 groups showed dose-dependently reduced ROS levels compared to the OVA group.

#### Effect of GTE on pulmonary inflammation and mucus production in lung tissue of asthmatic mice

**Figure 2:**
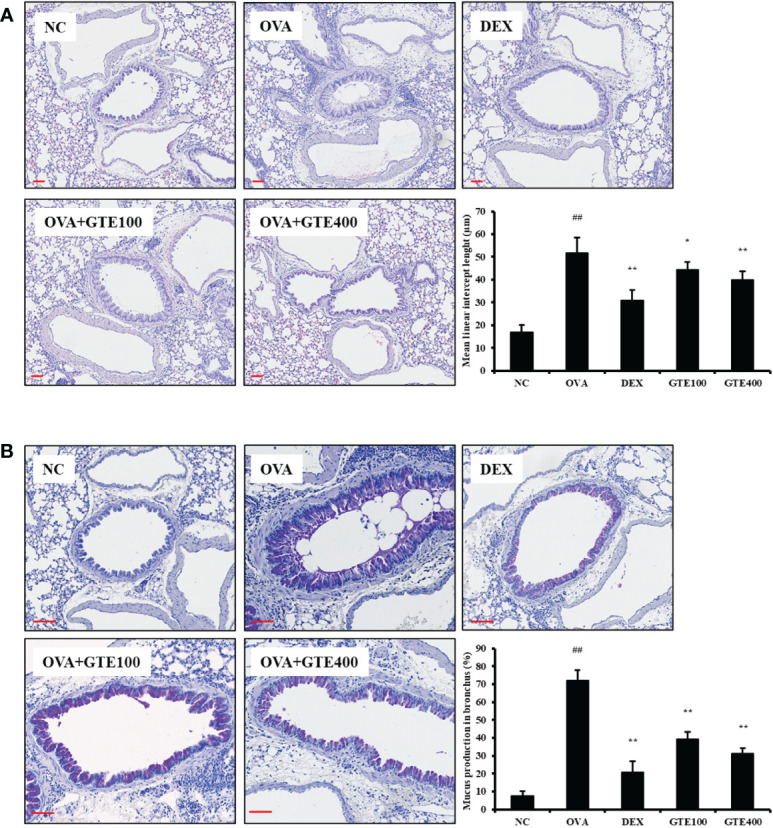
Green tea extract (GTE) inhibits the inflammation **(A)** and mucus overproduction **(B)** in lungs after ovalbumin (OVA) administration. Values are shown as means ± SD (*n* = 8). Significant differences: ^##^ *p* < 0.01 vs. NC; ^*, **^ *p* < 0.05 and *p* < 0.01 vs. OVA, respectively. Scale bar = 100 µm.

As shown in **Figure 2A**, H&E-stained lung sections of asthmatic mice showed an accumulation of inflammatory cells in peribronchiolar lesions compared to the NC group (*p* < 0.01). The lung tissues of DEX-treated mice showed a reduction in inflammatory cell infiltration caused by OVA exposure (*p* < 0.01). Similar to DEX-treated mice, GTE-treated mice (100 and 400 mg/kg/day) showed dose-dependent inhibition of inflammatory cell infiltration (*p* < 0.05 and *p* < 0.01, respectively). As indicated in **Figure 2B**, mucus overproduction (positive: stained purple) was significantly increased in the airways of OVA mice compared to that in NC mice (*p* < 0.01), whereas lung sections from GTE (100 and 400 mg/kg/day) mice showed dose-dependently decreased mucus production (*p* < 0.01) compared to that in OVA mice. The suppression of mucus production was similar to that observed in the DEX group.

#### Inhibitory effect of GTE on serum IgE and Th2 cytokines in BALF of OVA-challenged mice

**Figure 3:**
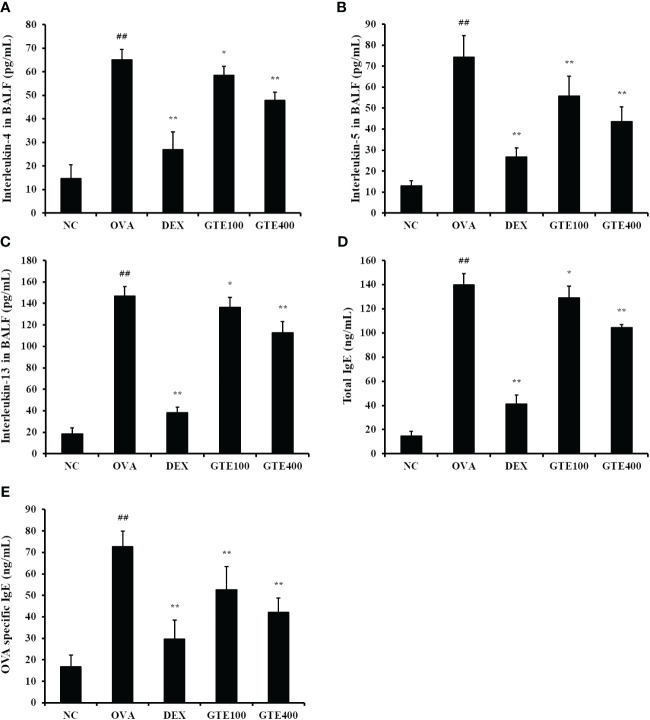
Green tea extract (GTE) inhibits the increased levels of interleukin (IL)-4 **(A)**, IL-5 **(B)**, IL-13 **(C)** in the bronchoalveolar lavage fluid (BALF), and total immunoglobulin **(Ig)** E **(D)** and ovalbumin (OVA)-specific IgE **(E)** in serum. Values are shown as means ± SD (*n* = 8). Significant differences: ^##^ *p* < 0.01 vs. NC; ^*, **^ *p* < 0.05 and *p* < 0.01 vs. OVA, respectively.

IL-4, IL‐5, and IL‐13 levels in the OVA group were significantly higher than those in the NC group (**Figures 3A–C**, *p* < 0.01). In contrast, administration of DEX (*p* < 0.01) or GTE (400 mg/kg/day; *p* < 0.01) reduced the cytokine levels compared to those in the OVA group. Compared to the NC group, the OVA group showed a marked elevation in OVA-specific IgE and total IgE in serum (**Figures 3D, E**; *p* < 0.01). However, DEX (*p* < 0.01) and GTE (400 mg/kg/day; *p* < 0.01) administration significantly reduced OVA-specific IgE and total IgE levels in the serum in a dose-dependent manner compared with the OVA group.

#### Effects of GTE on MMP-9 expression in lung tissue of OVA-induced asthmatic mice

**Figure 4:**
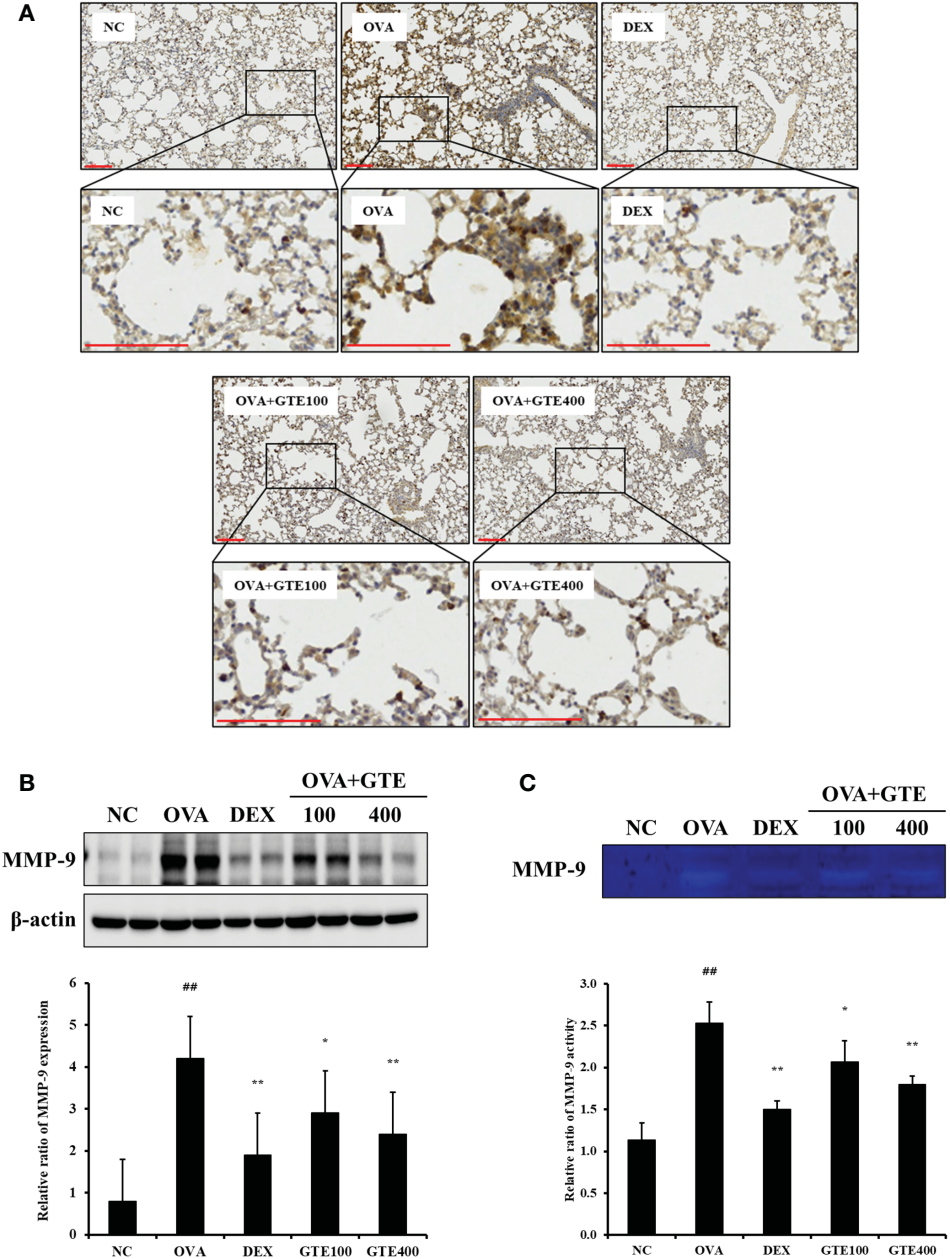
Green tea extract (GTE) reduces matrix metalloproteinase (MMP)-9 levels in lung tissue as confirmed by immunohistochemistry **(A)** and western blotting **(B)**. GTE alleviated MMP-9 activity as confirmed by zymography **(C)**. Values are shown as means ± SD (*n* = 8). Significant differences: ^##^ *p* < 0.01 vs. NC; ^*, **^ *p* < 0.05 and *p* < 0.01 vs. OVA, respectively. Scale bar = 100 µm.

To evaluate MMP‐9 protein expression levels, IHC staining and western blotting of lung tissues were performed (**Figures 4A, B**). As shown in **Figure 4B**, OVA-induced asthmatic mice showed a significantly higher level of MMP‐9 (*p* < 0.01) than normal control mice. In contrast, the GTE-treated mice (100 and 400 mg/kg/day) exhibited significantly lower level of MMP‐9 (*p* < 0.05 and *p* < 0.01, respectively) than the OVA mice. Gelatin zymography was performed to assess the MMP-9 activity (**Figure 4C**). Similar to the IHC and western blot results, the OVA group exhibited significantly higher MMP-9 activity (*p* < 0.01) than the control mice. However, GTE-treated mice (100 and 400 mg/kg/day) exhibited dose-dependently decreased the activity of MMP‐9 (*p* < 0.05 and *p* < 0.01) compared to the OVA mice.

#### Effects of GTE on the MAPKs inflammatory pathway

**Figure 5:**
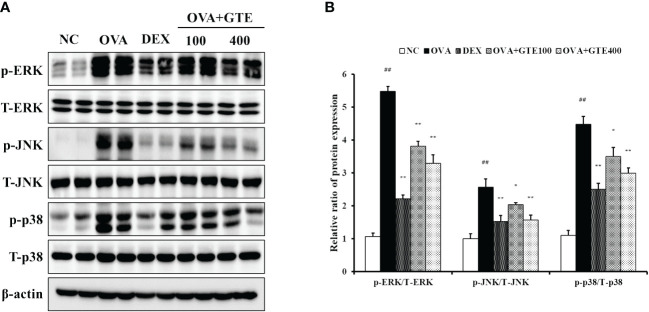
Green tea extract (GTE) suppresses ERK, JNK, and p38 phosphorylation **(A)**. Densitometric values were calculated using ChemiDoc **(B)**. Values are shown as means ± SD (*n* = 8). Significant differences: ^##^ *p* < 0.01 vs. NC; ^*, **^ *p* < 0.05 and *p* < 0.01 vs. OVA, respectively.

As indicated in **Figures 5A, B**, the OVA mice showed significantly increased phosphorylation of ERK, JNK, and p38 compared to normal control mice (*p* < 0.01). In contrast, the elevated MAPKs phosphorylation was significantly inhibited by DEX and 400 mg/kg/day GTE (*p* < 0.01).

#### Effect of GTE on antioxidant markers in the lung tissue of mice

**Figure 6:**
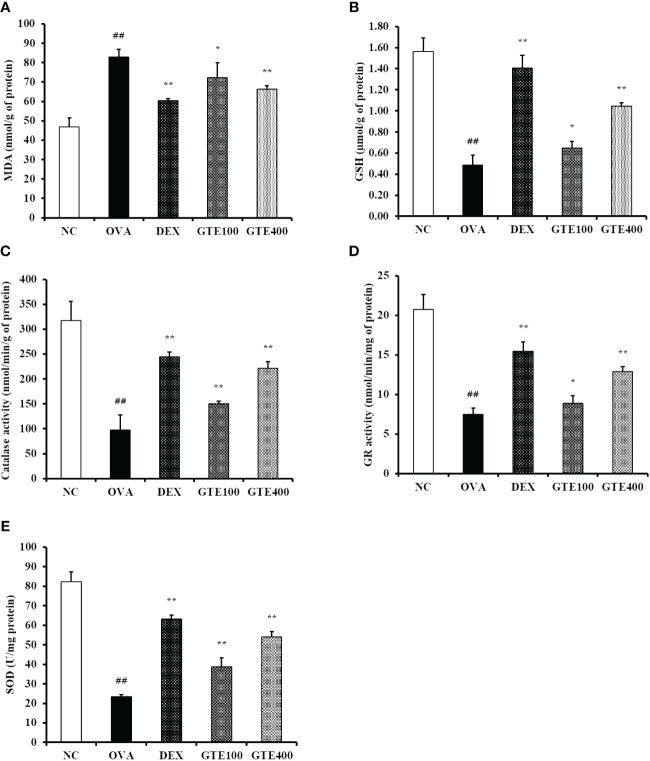
Green tea extract (GTE) reduces oxidative stress in lung tissue. **(A)** Malondialdehyde (MDA), **(B)** glutathione (GSH), **(C)** catalase (CAT), **(D)** glutathione reductase (GR), **(E)** superoxide dismutase (SOD) levels. Values are shown as means ± SD (*n* = 8). Significant differences: ^##^ *p* < 0.01 vs. NC; ^*, **^ *p* < 0.05 and *p* < 0.01 vs. OVA, respectively.

As shown in **Figures 6A–E**, the mean activities of CAT, GR, and GSH, and the mean SOD level in the lung tissue homogenates of the OVA group were significantly lower than those in the control group. The GTE400 group showed significantly elevated CAT, GR, and SOD activities, and GSH level compared to the OVA group (*p* < 0.01). In contrast, elevated MDA level observed in the OVA group was dose-dependently decreased in the GTE100 and GTE400 groups, respectively (*p* < 0.05 and *p* < 0.01).

### *In vitro* experiments

#### Effect of GTE on mRNA expression of proinflammatory cytokines in LPS-stimulated NCI-H292 cells

**Figure 7:**
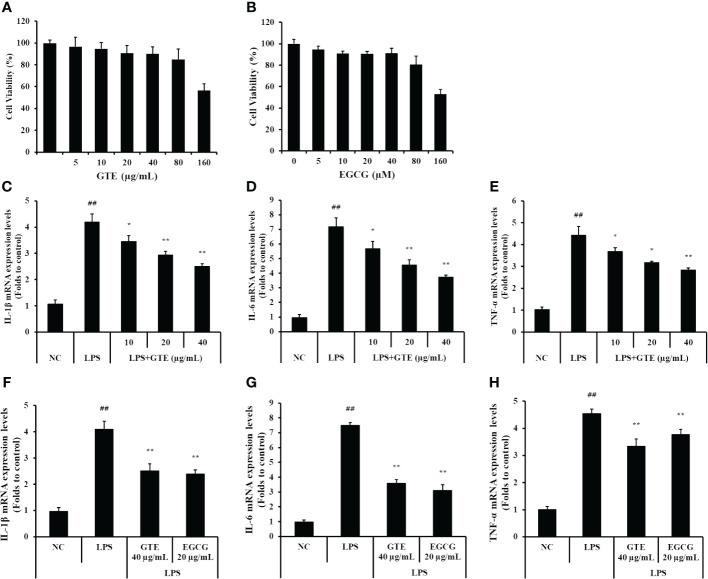
The concentration of **(A)** green tea extract (GTE) and **(B)** epigallocatechin gallate (EGCG) used in the experiment was determined based on the cytotoxicity results in NCI-H292 cells. GTE and EGCG reduced the increased levels of **(C, F)** interleukin (IL)-1β, **(D, G)** IL-6, **(E, H)** tumor necrosis factor (TNF)-α in the lipopolysaccharide (LPS)-stimulated cells. Cells were pretreated with 10, 20, and 40 µg/mL of GTE or 20 µg/mL of EGCG before LPS (0.5 µg/mL) treatment. Values are shown as means ± SD (*n* = 3). Significant differences: ^##^ *p* < 0.01 vs. NC; ^*, **^ *p* < 0.05 and *p* < 0.01 vs. LPS, respectively.

GTE and EGCG had no cytotoxic effect to NCI-H292 cells at concentrations up to 80 μg/mL (**Figures 7A, B**). Therefore, up to 40 μg/mL of GTE and 20 μg/mL of EGCG were used in subsequent experiments. LPS-treated cells had statistically elevated mRNA levels of TNF-α, IL-1β, and IL-6 compared to untreated cells (**Figures 7C–H**, *p* < 0.01). However, LPS-stimulated mRNA expressions of these proinflammatory cytokines were dose-dependently decreased by treatment with GTE (**Figures 7C–E**). Also, LPS-stimulated mRNA expression of these proinflammatory cytokines was significantly decreased by treatment with 20 μg/mL of EGCG (**Figures 7F–H**, *p* < 0.01).

#### Effects of GTE on the MAPKs pathway in LPS-treated NCI-H292 cells

**Figure 8:**
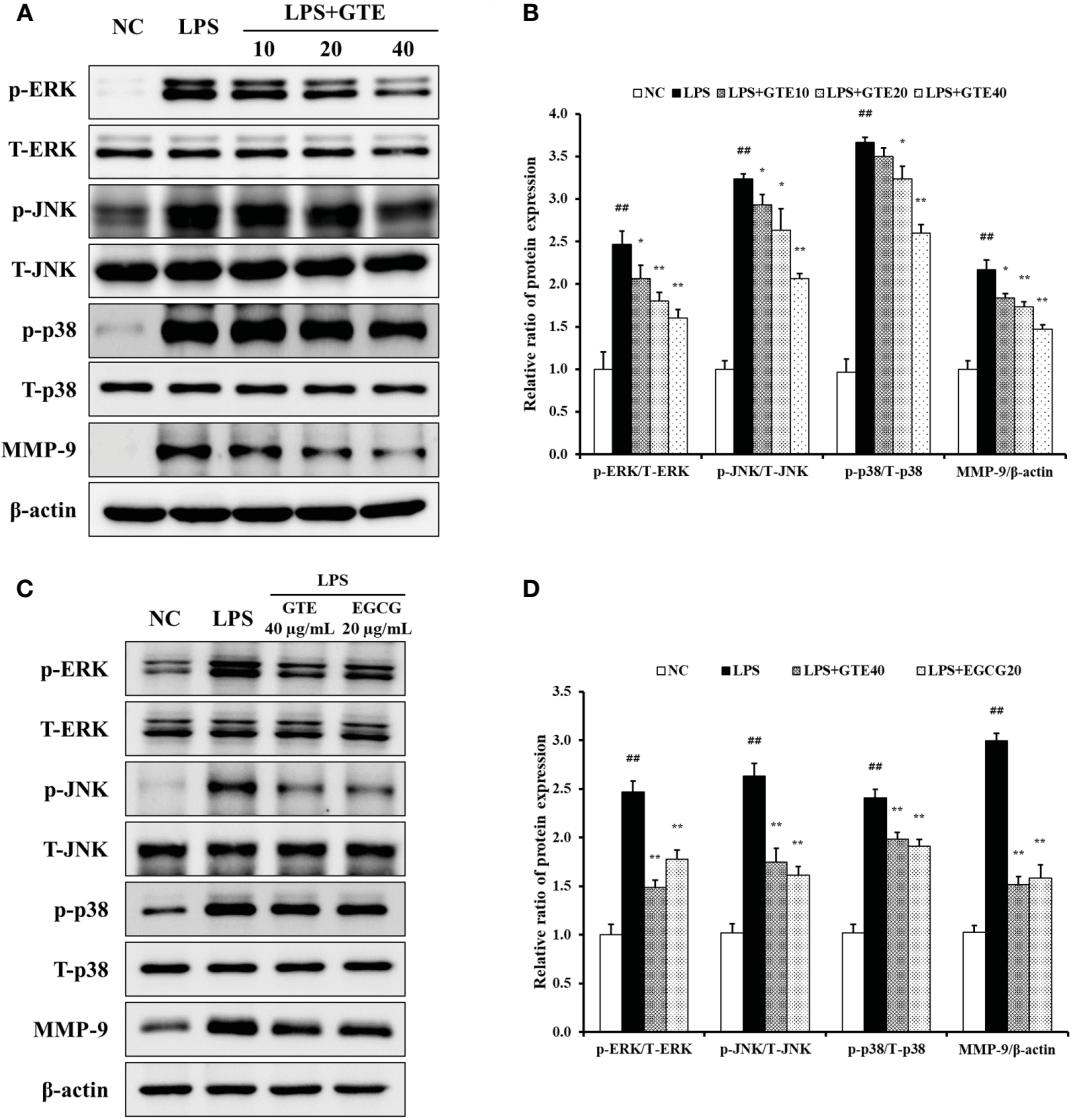
Green tea extract (GTE) inhibits dose-dependently increased ERK, JNK, and p38 phosphorylation and matrix metalloproteinase (MMP)-9 expression **(A)**. GTE (40 µg/mL) and epigallocatechin gallate (EGCG, 20 μg/mL) suppress increased ERK, JNK, and p38 phosphorylation and matrix metalloproteinase (MMP)-9 expression **(C)**. Densitometric values were calculated using ChemiDoc **(B, D)**. Cells were pretreated with 10, 20, and 40 µg/mL of GTE before LPS (0.5 µg/mL) treatment. Values are shown as means ± SD (*n* = 3). Significant differences: ^##^ *p* < 0.01 vs. NC; ^*, **^ *p* < 0.05 and *p* < 0.01 vs. LPS, respectively.

**Figure 9:**
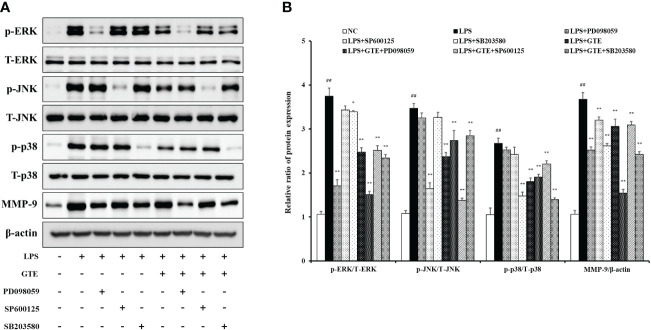
Effects of green tea extract (GTE) on the mitogen-activated protein kinase (MAPK) pathway in terms of matrix metalloproteinase (MMP)-9 production in lipopolysaccharide (LPS)-stimulated NCI-H292 cells. Cells were pretreated with 40 µg/mL of GTE, and 10 µM of PD098059, SP600125, and SB203680 before LPS (0.5 µg/mL) treatment **(A)**. Densitometric values were calculated using ChemiDoc **(B)**. Values are shown as means ± SD (*n* = 3). Significant differences: ^##^ *p* < 0.01 vs. NC; ^*, **^ *p* < 0.05 and *p* < 0.01 vs. LPS, respectively.

LPS-treated cells showed elevated phosphorylation of MAPKs compared to untreated cells (**Figures 8A, B**). However, GTE-treated cells (40 μg/mL) showed a significant reduction of MAPKs phosphorylation compared with LPS-treated cells (*p* < 0.01). The expression pattern of MMP-9 was similar to that of MAPKs. LPS-treated cells showed a significant increase in MMP-9 expression, whereas GTE-treated cells showed a dose-dependent decrease compared to LPS-stimulated cells. GTE (40 μg/mL) and EGCG (20 μg/mL) showed similar results on the expression of MAPKs and MMP-9 (**Figures 8C, D**). Treatment of NCI-H292 cells with GTE and MAPKs inhibitors significantly reduced MAPKs phosphorylation compared to that in NCI-H292 cells treated with only MAPKs inhibitors (**Figures 9A. B**).

## Discussion

Asthma is a chronic pulmonary disease that causes various symptoms, such as respiratory inflammation, excessive mucus secretion, and AHR (19). In this study, we examined the therapeutic effects of GTE treatment in an OVA-induced mouse model of asthma, focusing on the oxidative stress-mediated inflammatory response and its underlying mechanisms. GTE effectively decreased proinflammatory cell counts and cytokine levels in the BALF and reduced OVA-specific IgE production in the serum. These preventive effects corresponded to a reduction in AHR and mucus production in the lungs of OVA-challenged mice. Furthermore, GTE treatment notably improved antioxidant enzyme activities followed by suppressed phosphorylation of JNK, ERK, p38 and expression of MMP‐9 increased by OVA inhalation.

Th2 cytokines are major contributors to the pathogenesis of allergic diseases, including asthma (20). Numerous clinical and preclinical studies have demonstrated a relationship between Th2 lymphocytes and the pathogenesis of asthma, including mucus hypersecretion and inflammation (3). In current study, GTE significantly suppressed the levels of Th2 cytokines, OVA‐specific IgE in serum, and inflammatory cell counts in BALF, and these effects of GTE corresponded with the histological findings in lung tissue, such as decreased inflammatory response and mucus secretion. These findings indicated that GTE significantly inhibited the pathogenesis of asthma by reducing Th2 cytokine levels.

The MAPKs pathway plays a key role in the downstream signaling events that lead to inflammation (21). Several studies have shown that phosphorylation of MAPKs triggers inflammatory and immune responses by regulating the production of TNF-α, IL-6, and other inflammatory factors in asthma (22). Previous studies have shown that ROS production causes cellular oxidative stress, leading to the activation of MAPKs. In particular, Bao and collaborators (23) reported that ozone-driven oxidative stress induced phosphorylation p-38 in an allergic asthma model, and Ma and collaborators (24) confirmed that oxidative stress activated MAPKs signaling in an OVA-challenged mouse model of asthma. These findings strongly support the hypothesis that the pathogenicity of asthma involves oxidative stress-driven MAPKs phosphorylation. In this study, administration of GTE elevated GSH levels and anti-oxidative enzyme activities, and reduced MDA levels, a marker of lipid peroxidation. These effects were followed by a dose-dependent reduction in MAPKs phosphorylation in OVA-induced asthmatic mice. Therefore, reduced oxidative stress and MAPKs signaling may be the mechanisms underlying the preventive effects of GTE on asthma development.

MMP‐9 belongs to the extracellular protease family and plays a key role in airway remodeling, including extracellular matrix reorganization, regulation of protein degradation, and cell migration (25, 26). The activation of MMP-9 is closely associated with the phosphorylation of MAPKs, which occurs in an asthmatic response (7). Lim and collaborators (27) reported that phosphorylation of MAPKs activated MMP-9 expression in OVA-challenged mice, and that MMP-9 levels were higher in samples from patients with allergic asthma, which were eliminated by MAPKs inhibitors (28). In the current study, the administration of GTE significantly suppressed the levels and activity of MMP‐9 as showed by IHC, western blotting, and zymography. These findings indicated that GTE reduced Th2 cytokine release and inhibited MMP-9 levels by decreasing MAPKs phosphorylation.

The protective effect of GTE in asthma is considered to be related with ROS production. Green tea has been studied for its antioxidant activity in various lung diseases (29–31). GTE reduces GSH, SOD and CAT levels in the lungs of a cigarette smoke-induced lung injury rat model (29, 30). Also, GTE suppresses paraquat-induced lung fibrosis in mouse via inhibiting nitric oxide production (31). Especially, polyphenols and catechins in GTE have a strong antioxidant effect, and polysaccharides also have a dose-dependent superoxide scavenging activity (32). These studies strongly support our results.

In conclusion, GTE treatment significantly suppressed Th2 cytokine production and inflammation in OVA-induced asthmatic mice and human lung epithelial cells. This effect of GTE was associated with the inhibition of oxidative stress-mediated MAPKs phosphorylation and MMP-9 inhibition. Therefore, the findings of this study suggest that GTE could be a potential therapeutic supplement for allergic asthma.

## Data availability statement

The original contributions presented in the study are included in the article/**Supplementary Material**., further inquiries can be directed to the corresponding author/s.

## Ethics statement

The animal study was approved by Animal Experiments of Chungnam National University. The study was conducted in accordance with the local legislation and institutional requirements.

## Author contributions

J-WKi: Investigation, Writing – original draft. J-HK: Formal analysis, Writing – review & editing. J-SJ: Formal analysis, Writing – review & editing. C-YK: Formal analysis, Writing – review & editing. E-HC: Formal analysis, Writing – review & editing. E-JH: Writing – review & editing. H-JK: Writing – review & editing. S-HK: Writing – review & editing. J-WKo: Conceptualization, Funding acquisition, Supervision, Writing – review & editing. T-WK: Conceptualization, Funding acquisition, Supervision, Writing – review & editing.
