# Supplementary figures and images for: Green tea extract suppresses airway inflammation via oxidative stress-driven MAPKs/MMP-9 signaling in asthmatic mice and human airway epithelial cells
Source: Front Immunol. 2024 Apr 30;15:1362404. doi: 10.3389/fimmu.2024.1362404 (PMC11091254; doi:10.3389/fimmu.2024.1362404)

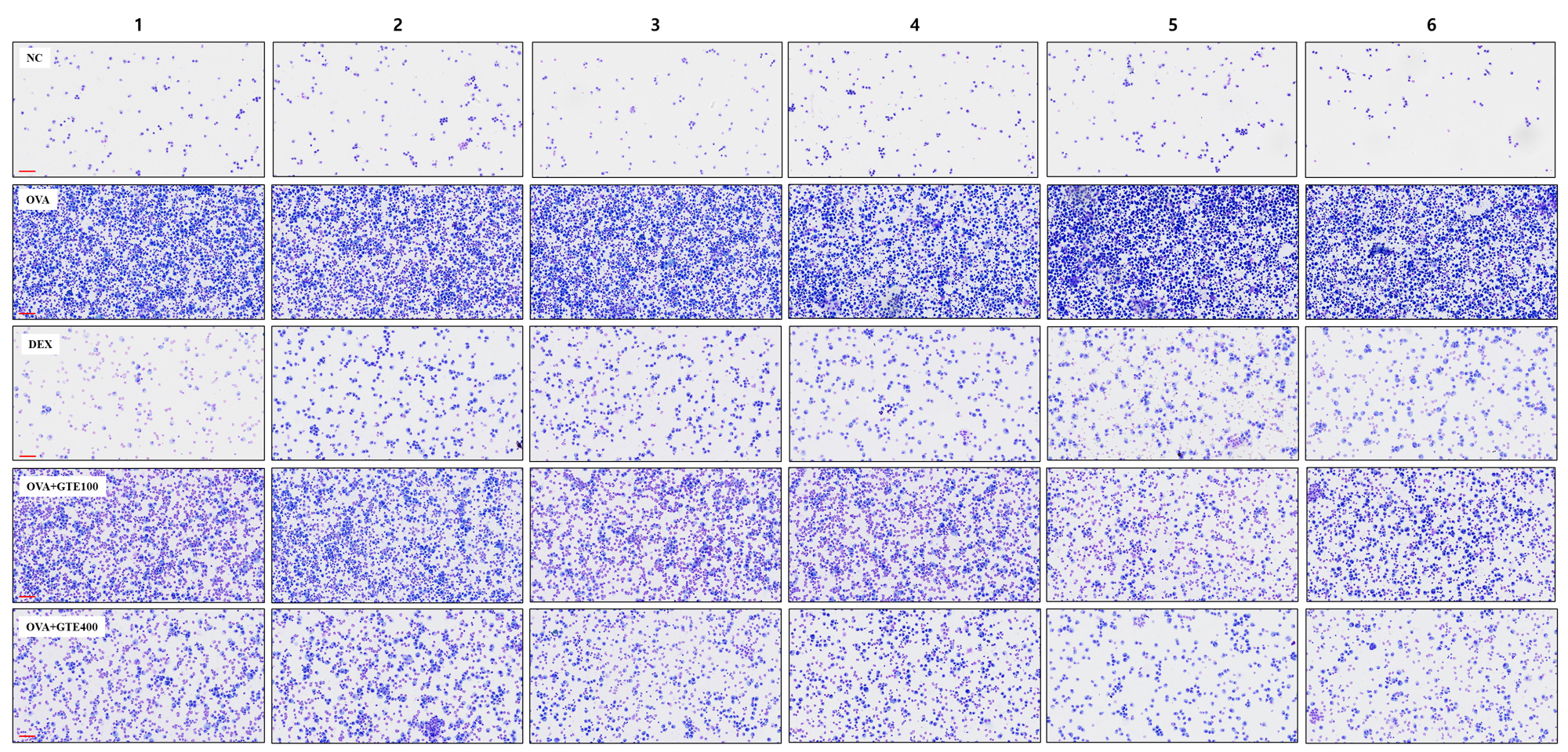

Supplement: Supplementary file 2 [file DataSheet_2.zip › Supplementary figure/Original file shown in Fig.1B.tif]

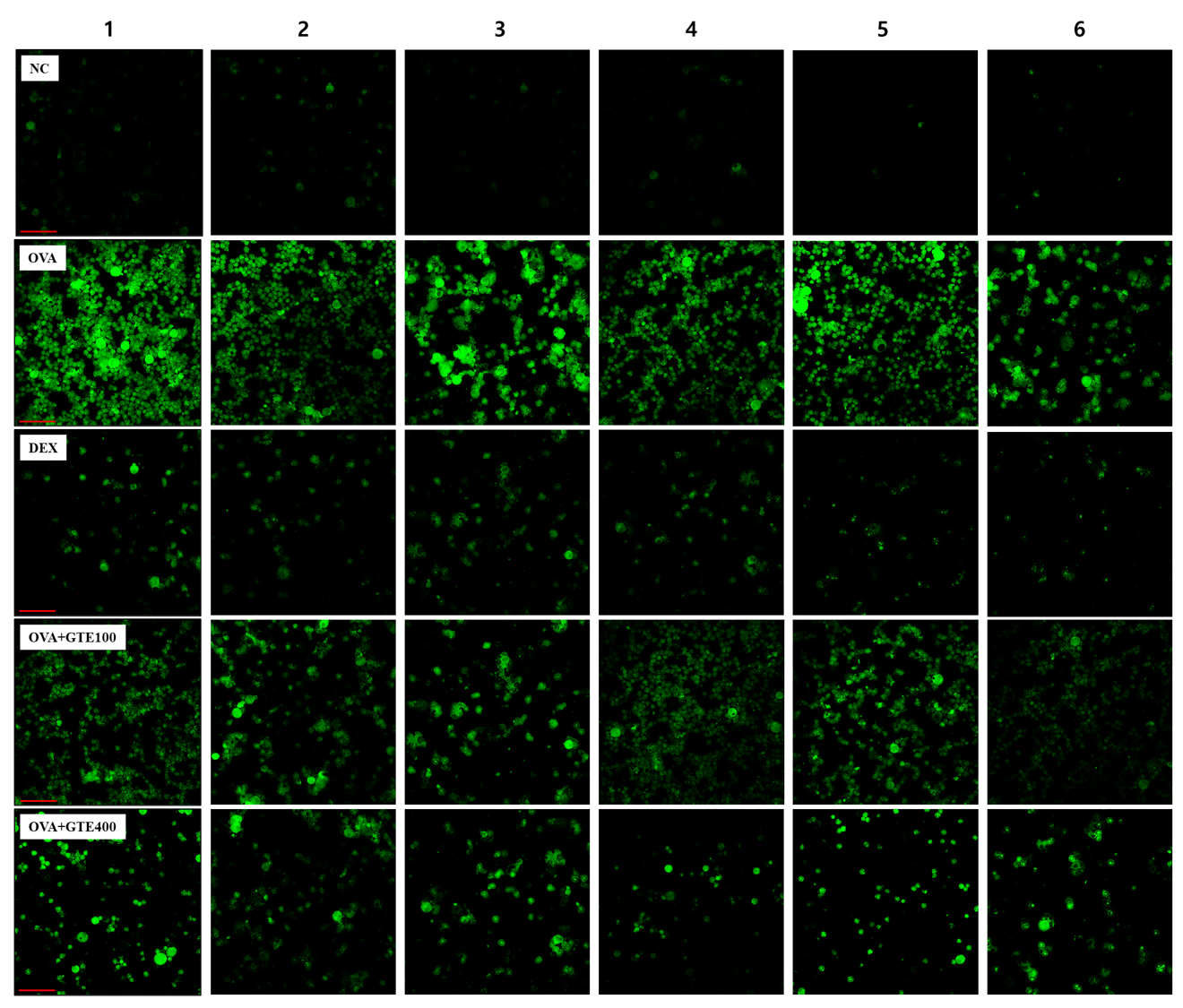

Supplement: Supplementary file 2 [file DataSheet_2.zip › Supplementary figure/Original file shown in Fig.1C.tif]

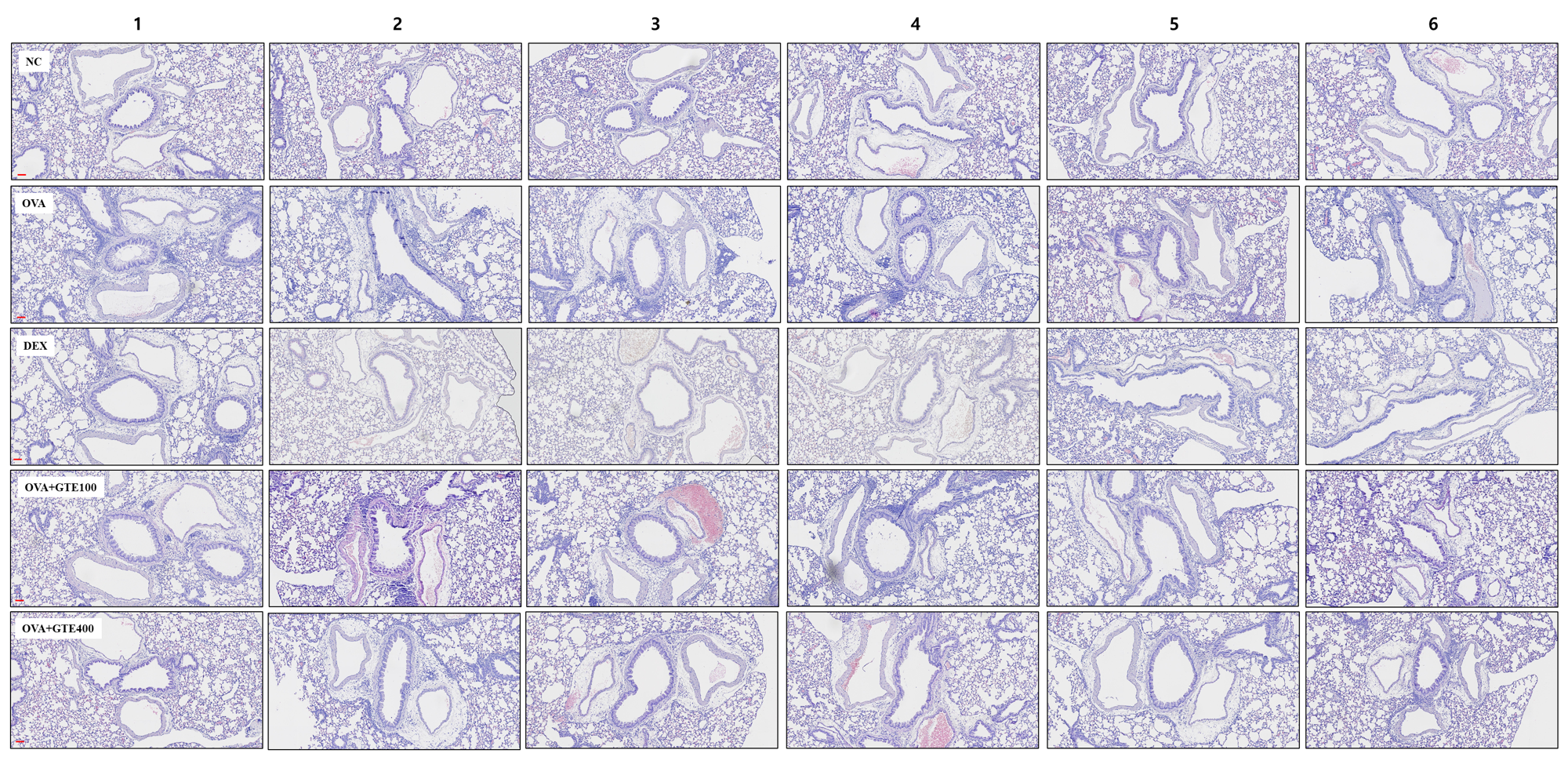

Supplement: Supplementary file 2 [file DataSheet_2.zip › Supplementary figure/Original file shown in Fig.2A.tif]

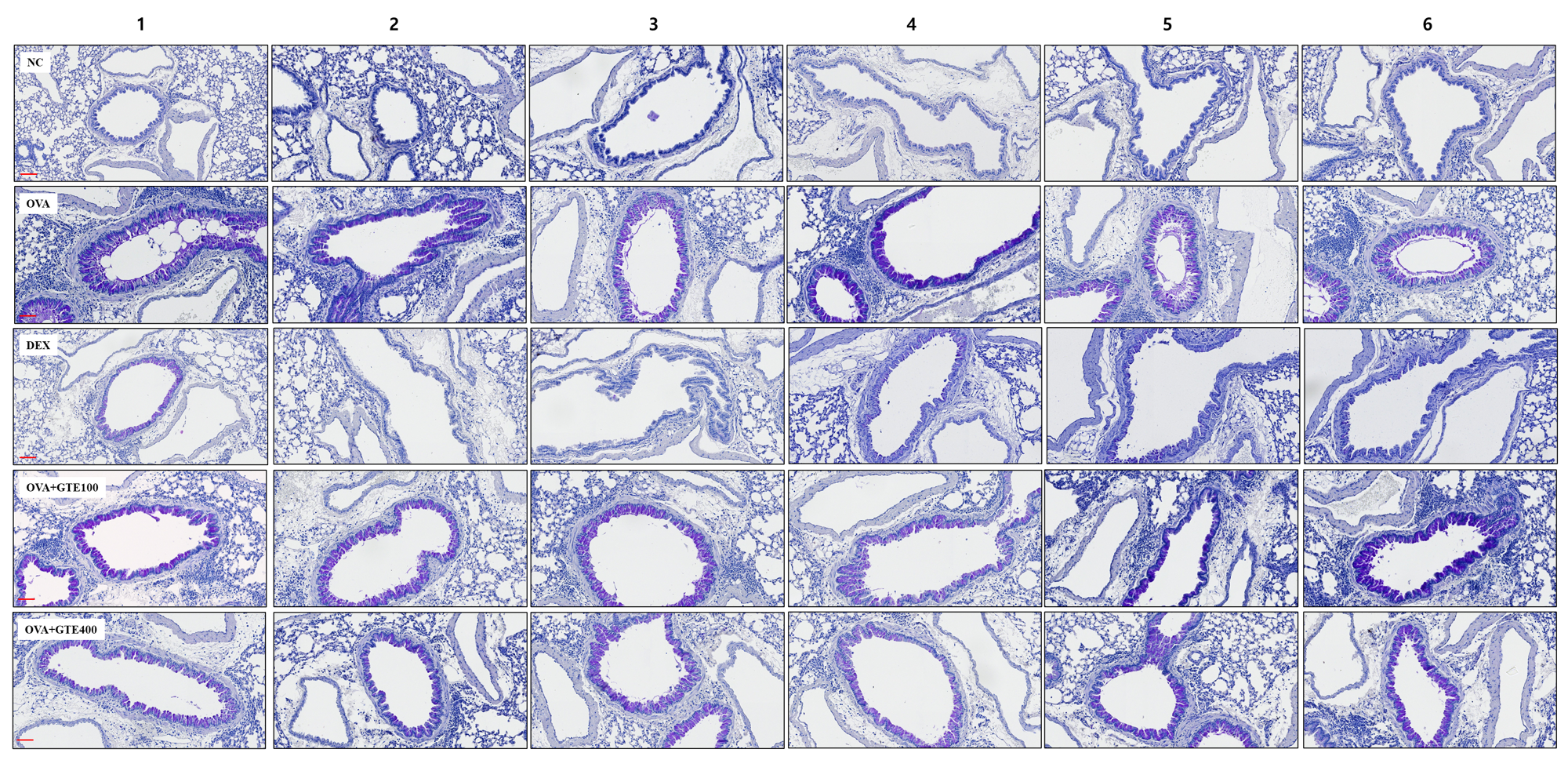

Supplement: Supplementary file 2 [file DataSheet_2.zip › Supplementary figure/Original file shown in Fig.2B.tif]

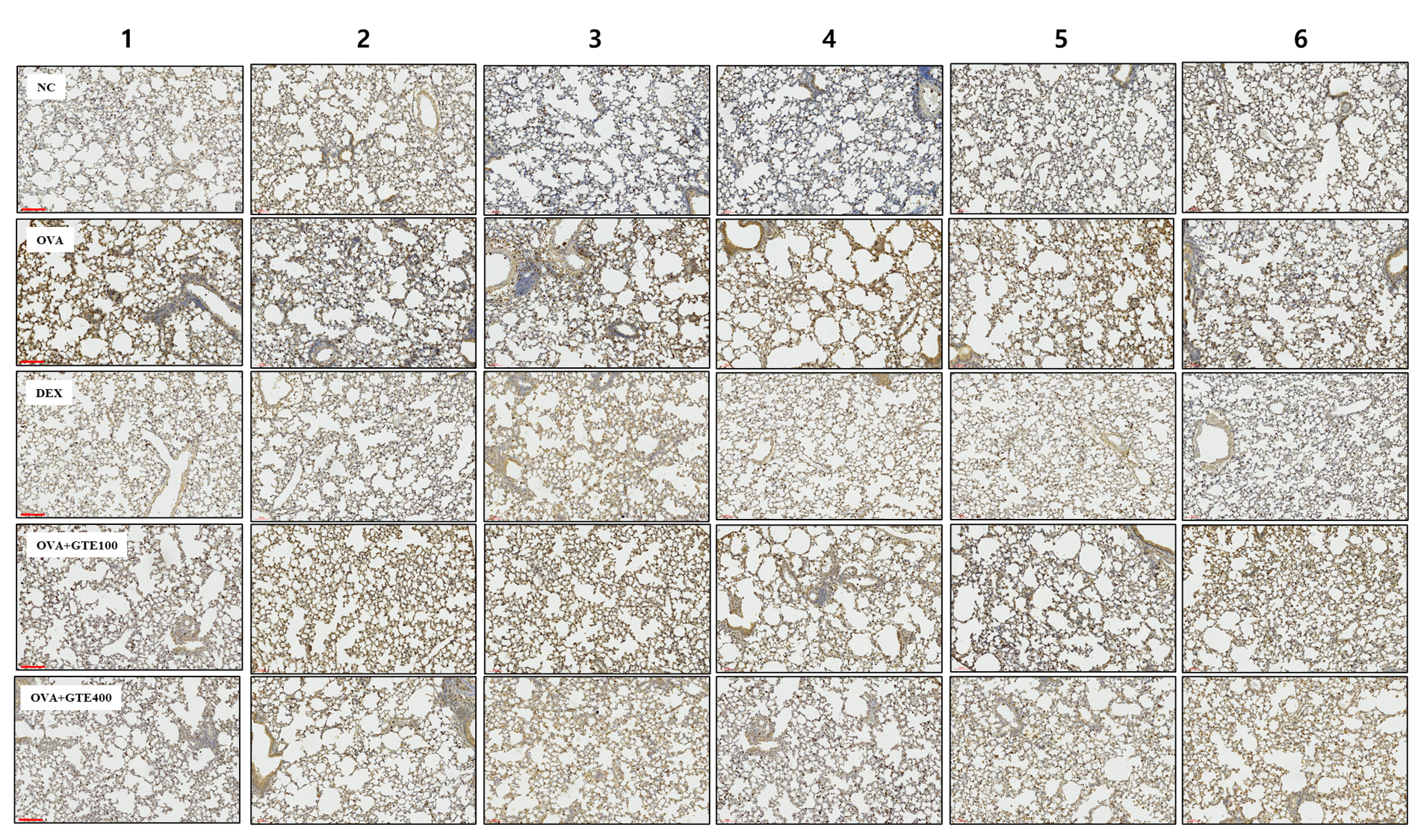

Supplement: Supplementary file 2 [file DataSheet_2.zip › Supplementary figure/Original file shown in Fig.4A.tif]

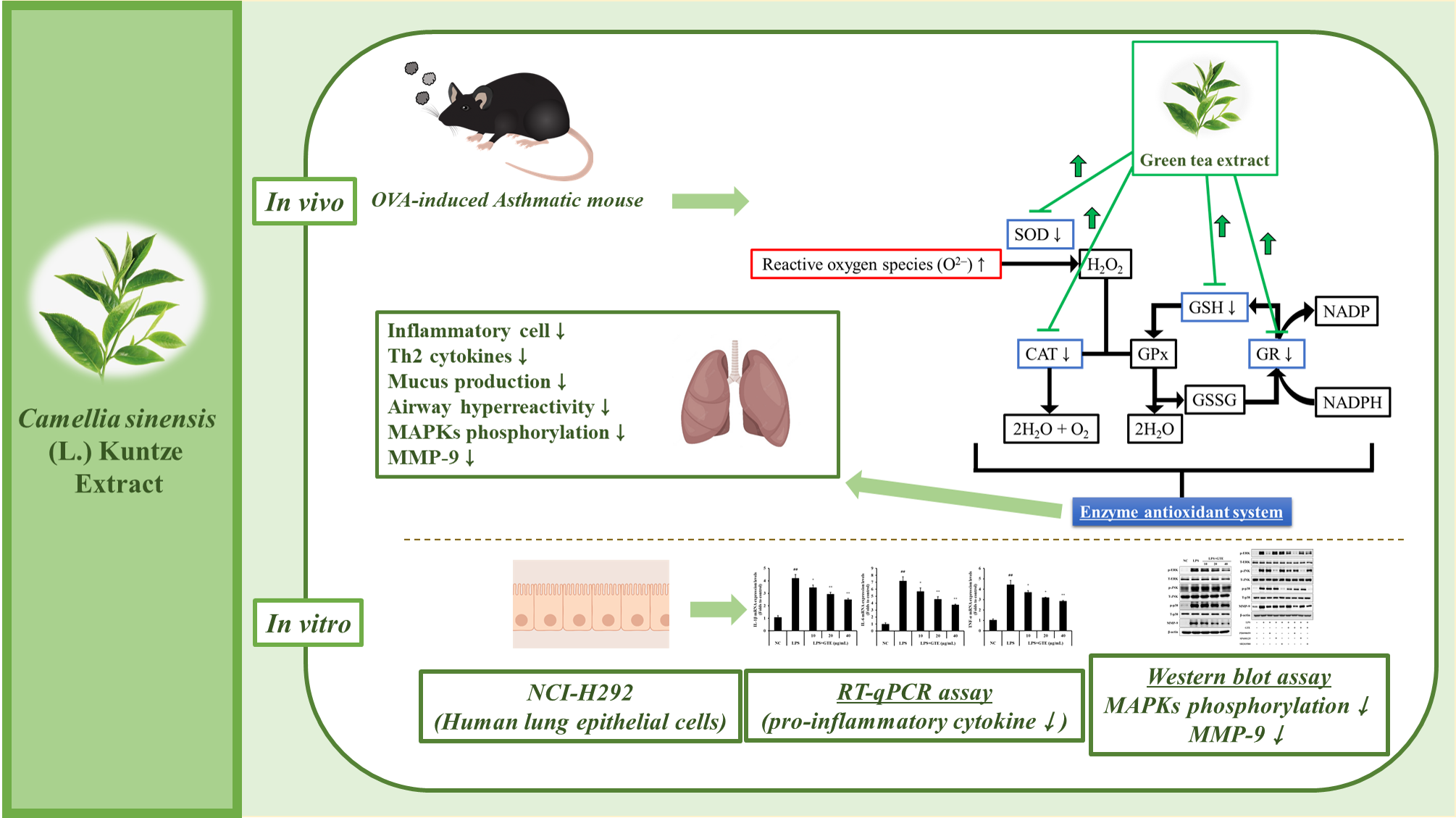

Supplement: Supplementary file 4 [file Image_1.tif]
